# What every intensivist should know about Tocilizumab

**DOI:** 10.1186/s13054-021-03696-1

**Published:** 2021-07-27

**Authors:** Daniel Andrea Hofmaenner, Pedro David Wendel Garcia, Christoph Camille Ganter, Silvio D. Brugger, Philipp Karl Buehler, Sascha David

**Affiliations:** 1 Institute of Intensive Care Medicine, University Hospital Zurich, University of Zurich, Rämistrasse 100, 8091 Zurich, Switzerland; 2 Department of Infectious Diseases and Hospital Epidemiology, University Hospital Zurich, University of Zurich, Zurich, Switzerland

Treatment strategies against corona virus disease (COVID)-19 have been investigated since the recognition of SARS-CoV-2. Anti-viral strategies such as Remdesivir have shown effects in the early disease phase [1]. Progression is often driven by a dysregulated host response triggering a hyper-inflammatory phenotype. In this state, anti-infectives were inferior to anti-inflammatory strategies. Dexamethasone improves survival in COVID-19 patients with pneumonia requiring oxygen [2]. Tocilizumab—a humanized anti-human IL-6 receptor (IL-6R) antibody of the IgG1 subclass—has gained attention due to a potential synergistic survival benefit [3], especially in certain subpopulations. Tocilizumab is no stranger to rheumatologists as it has widely been used to treat various disorders such as rheumatoid arthritis.

Although some intensivists had experience with Tocilizumab to control CAR-T-associated cytokine release syndrome (CRS), it remains an alien in the intensivists’ armamentarium. Still, many colleagues have adopted this drug in light of the recent data to treat severe COVID-19. Given its unknown biological specifics in the critical care context, we have analyzed the longitudinal course of IL-6, together with C-reactive protein (CRP), procalcitonin (PCT) and leucocyte counts in 16 COVID-19 patients (Fig. 1). Baseline characteristics and outcomes are demonstrated in Table 1. Tocilizumab was almost exclusively administered if there was a progression of the disease (i.e., requirement of invasive ventilation in those on high-flow oxygen or deterioration in invasively ventilated patients) despite prior steroid use. Aside from reported side effects [3], we want to highlight the following phenomena:

1. *Increase in IL-6* Circulating IL-6 serum levels increase rapidly and profoundly (up to 38×), peak around day 3–5 and stay elevated for many days after Tocilizumab administration. Comparable increases have also been described in other studies [4].
2. *Suppression of CRP* In line with the previous literature [4], IL-6R blockade leads to a sustained suppression of downstream effectors such as CRP. In our cohort, this effect was observed for approximately 14 days rendering its clinical use as a biomarker of infection useless (“CRP-blind spot”).
3. *Unchanged leucocyte count and PCT* Despite their limited sensitivity and specificity, leukocyte count and PCT are rather unaffected by Tocilizumab and might give additional information during the “*CRP-blind spot”*. However, the relevance of these phenomena has still not been elucidated in COVID-19 and should be seen in an individual context.

Additionally, we want to highlight two aspects that are of importance when administering Tocilizumab:

4. *Increased infection risk* Blockade of the IL-6R increases the risk of serious infections and should not be used in sepsis. Bacterial, viral and opportunistic infections have been reported [5].
5. *Development/aggravation of encephalopathy* Due to its pharmacodynamics, Tocilizumab is unable to cross the blood–brain-barrier but increases the circulating amount of IL-6 (a small molecule that can easily do so) up to 3800%. This phenomenon of induced encephalopathy is known from CAR-T-associated CRS and should be considered when giving Tocilizumab to awake patients (particularly in the context of delirium) [6]. Whereas it has been used in the CAR-T context according to the standard algorithm usually before steroids, in COVID-19, Tocilizumab’s effect might be different after prior steroid use.

**Fig. 1:**
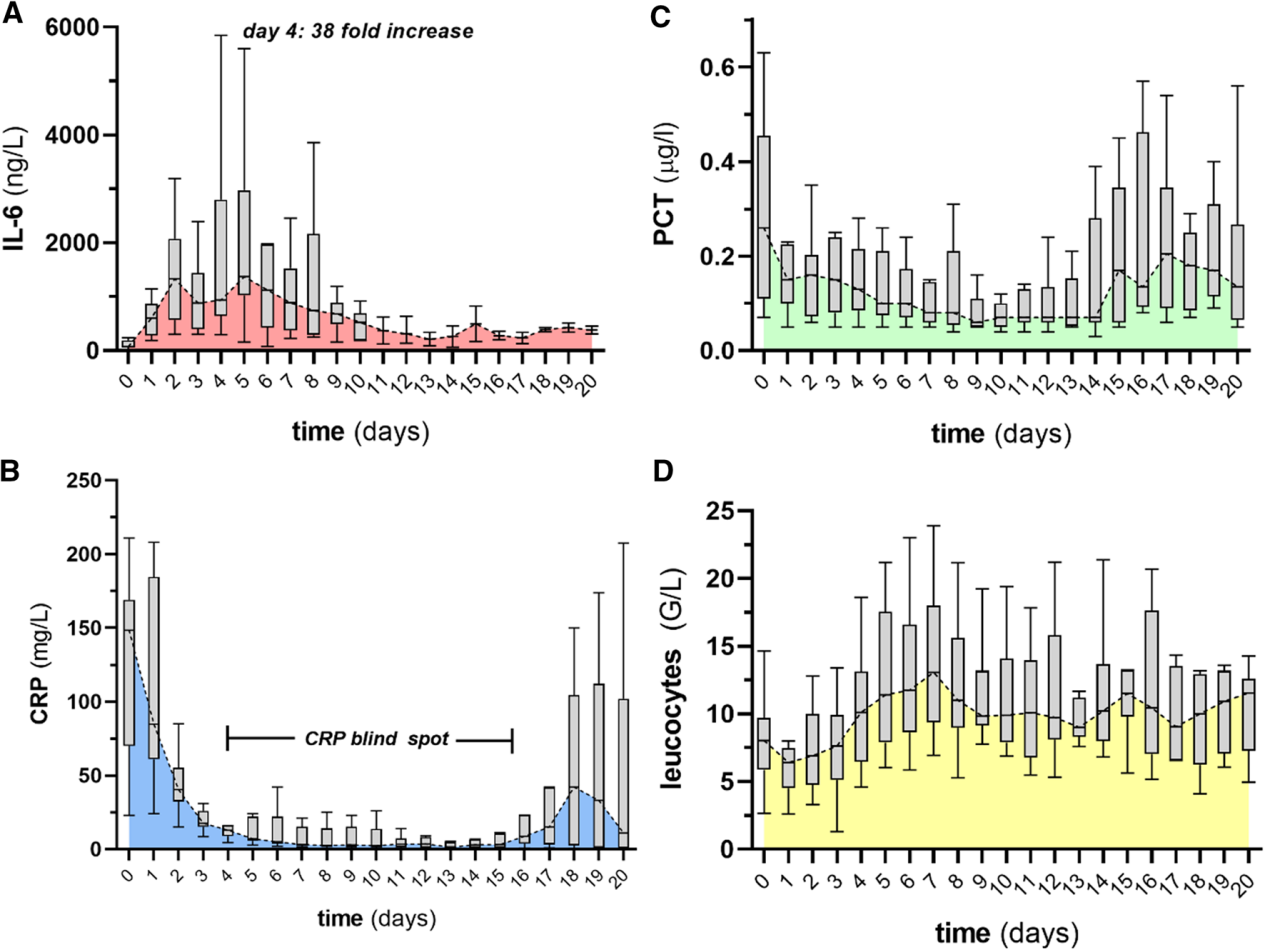
Longitudinal course over 20 days after Tocilizumab administration in 16 critically ill COVID-19 patients (local ethical approval: 2020-00646). Box and whiskers blots together with colored area demonstrate circulating levels of **a** Interleukin (IL)-6, **b** C-reactive protein (CRP), **c** procalcitonin (PCT) and **d** leucocytes. Days 0 = Tocilizumab administration (8 mg/kg bodyweight, max: 800 mg)

**Table 1:** Baseline characteristics and outcome of 16 critically ill COVID-19 patients

Age (years)	55.5 [47–63] (48–63)
BMI (kg/m^2^)	30.5 [26–33] (27–32)
Male gender	7/16 (43.8%)
*Comorbidities*	
Arterial hypertension	5/16 (31.3%)
CAD	1/16 (6.3%)
COPD	1/16 (6.3%)
Diabetes mellitus	4/16 (25%)
Chronic kidney disease	3/16 (18.8%)
SOFA score at ICU admission	11 [06–12] (11–12)
Invasive mechanical ventilation	13/16 (81.3%)
ICU survival	9/16 (56.3%)
Length of ICU stay (d)	26 [18–31] (19–32)
Steroids before Tocilizumab administration	13/16 (81.3%)

Results provided as absolute numbers and (percentages) or as median, [interquartile range] and (95% confidence interval), as appropriate

*BMI* body mass index,* CAD* coronary artery disease,* COPD* chronic obstructive pulmonary disease,* SOFA score* sequential organ failure assessment score, ICU; intensive care unit

Mechanistically, it has been proposed that the increase in IL-6 is the result of IL-6R blockade, inhibiting internalization of IL-6 after ligation with its receptor. In other words, the blocked IL-6R liberates the release of accumulated IL-6 into the circulation. One can speculate that a given IL-6 increase reflects its local production in the inflamed lung and that this increase might even be useful to predict a clinical Tocilizumab response. In our rather small cohort, no differences between survivors and non-survivors were detectable, but a controlled trial would be desirable.

## Data Availability

All data supports results for this comment are available with the corresponding author.

## References

[1] Paladugu S, Donato AA (2020). Remdesivir improved time to recovery in adults hospitalized with COVID-19 and lower respiratory tract involvement. Ann Intern Med.

[2] Horby P, Lim WS, Emberson JR, Mafham M, Bell JL, RECOVERY Collaborative Group (2021). Dexamethasone in hospitalized patients with Covid-19. N Engl J Med.

[3] Gordon AC, Mouncey PR, Al-Beidh F, Rowan KM, Nichol AD, REMAP-CAP Investigators (2021). Interleukin-6 receptor antagonists in critically ill patients with Covid-19. N Engl J Med.

[4] Keske Ş, Tekin S, Sait B, İrkören P, Kapmaz M, Çimen C (2020). Appropriate use of tocilizumab in COVID-19 infection. Int J Infect Dis.

[5] Pawar A, Desai RJ, Solomon DH, Santiago Ortiz AJ, Gale S, Bao M (2019). Risk of serious infections in tocilizumab versus other biologic drugs in patients with rheumatoid arthritis: a multidatabase cohort study. Ann Rheum Dis.

[6] Brudno JN, Kochenderfer JN (2019). Recent advances in CAR T-cell toxicity: mechanisms, manifestations and management. Blood Rev.

